# Supplementary material for: Thermal suppression of gametogenesis can explain historical collapses in larval recruitment in Strongylocentrotus purpuratus
Source: Commun Biol. 2025 Nov 3;8:1490. doi: 10.1038/s42003-025-08829-8 (PMC12583514; doi:10.1038/s42003-025-08829-8)
Supplement: Supplementary file 1 — Reporting Summary [file 42003_2025_8829_MOESM1_ESM.pdf]

nature portfolio

Corresponding author(s): Daniel K. Okamoto

Last updated by author(s): 2025/08/15

# Reporting Summary

Nature Portfolio wishes to improve the reproducibility of the work that we publish. This form provides structure for consistency and transparency in reporting. For further information on Nature Portfolio policies, see our Editorial Policies and the Editorial Policy Checklist.

## Statistics

For all statistical analyses, confirm that the following items are present in the figure legend, table legend, main text, or Methods section.

| n/a | Confirmed | |
|---|---|---|
| ☐ | ☒ | The exact sample size (*n*) for each experimental group/condition, given as a discrete number and unit of measurement |
| ☐ | ☒ | A statement on whether measurements were taken from distinct samples or whether the same sample was measured repeatedly |
| ☐ | ☒ | The statistical test(s) used AND whether they are one- or two-sided<br>*Only common tests should be described solely by name; describe more complex techniques in the Methods section.* |
| ☐ | ☒ | A description of all covariates tested |
| ☐ | ☒ | A description of any assumptions or corrections, such as tests of normality and adjustment for multiple comparisons |
| ☐ | ☒ | A full description of the statistical parameters including central tendency (e.g. means) or other basic estimates (e.g. regression coefficient) AND variation (e.g. standard deviation) or associated estimates of uncertainty (e.g. confidence intervals) |
| ☐ | ☒ | For null hypothesis testing, the test statistic (e.g. *F*, *t*, *r*) with confidence intervals, effect sizes, degrees of freedom and *P* value noted<br>*Give P values as exact values whenever suitable.* |
| ☐ | ☒ | For Bayesian analysis, information on the choice of priors and Markov chain Monte Carlo settings |
| ☐ | ☒ | For hierarchical and complex designs, identification of the appropriate level for tests and full reporting of outcomes |
| ☒ | ☐ | Estimates of effect sizes (e.g. Cohen's *d*, Pearson's *r*), indicating how they were calculated |

*Our web collection on statistics for biologists contains articles on many of the points above.*

## Software and code

Policy information about availability of computer code

| | |
|---|---|
| Data collection | no software was used to collect data |
| Data analysis | We used R (Version 4.5.0) Stan (Version 2.36), rstan (Version 2.32.2), brms (Version 2.22.0) , and bridgesampling (1.1-2) |

For manuscripts utilizing custom algorithms or software that are central to the research but not yet described in published literature, software must be made available to editors and reviewers. We strongly encourage code deposition in a community repository (e.g. GitHub). See the Nature Portfolio guidelines for submitting code & software for further information.

## Data

Policy information about availability of data

All manuscripts must include a data availability statement. This statement should provide the following information, where applicable:

- Accession codes, unique identifiers, or web links for publicly available datasets
- A description of any restrictions on data availability
- For clinical datasets or third party data, please ensure that the statement adheres to our policy

Data and metadata from this study are available at https://www.bco-dmo.org/project/818918 and both data and analysis code are available at https://doi.org/10.5281/zenodo.15420518.

# Research involving human participants, their data, or biological material

Policy information about studies with human participants or human data. See also policy information about sex, gender (identity/presentation), and sexual orientation and race, ethnicity and racism.

| | |
|---|---|
| Reporting on sex and gender | *Use the terms sex (biological attribute) and gender (shaped by social and cultural circumstances) carefully in order to avoid confusing both terms. Indicate if findings apply to only one sex or gender; describe whether sex and gender were considered in study design; whether sex and/or gender was determined based on self-reporting or assigned and methods used. Provide in the source data disaggregated sex and gender data, where this information has been collected, and if consent has been obtained for sharing of individual-level data; provide overall numbers in this Reporting Summary. Please state if this information has not been collected. Report sex- and gender-based analyses where performed, justify reasons for lack of sex- and gender-based analysis.* |
| Reporting on race, ethnicity, or other socially relevant groupings | *Please specify the socially constructed or socially relevant categorization variable(s) used in your manuscript and explain why they were used. Please note that such variables should not be used as proxies for other socially constructed/relevant variables (for example, race or ethnicity should not be used as a proxy for socioeconomic status). Provide clear definitions of the relevant terms used, how they were provided (by the participants/respondents, the researchers, or third parties), and the method(s) used to classify people into the different categories (e.g. self-report, census or administrative data, social media data, etc.) Please provide details about how you controlled for confounding variables in your analyses.* |
| Population characteristics | *Describe the covariate-relevant population characteristics of the human research participants (e.g. age, genotypic information, past and current diagnosis and treatment categories). If you filled out the behavioural & social sciences study design questions and have nothing to add here, write "See above."* |
| Recruitment | *Describe how participants were recruited. Outline any potential self-selection bias or other biases that may be present and how these are likely to impact results.* |
| Ethics oversight | *Identify the organization(s) that approved the study protocol.* |

Note that full information on the approval of the study protocol must also be provided in the manuscript.

# Field-specific reporting

Please select the one below that is the best fit for your research. If you are not sure, read the appropriate sections before making your selection.

☐ Life sciences ☐ Behavioural & social sciences ☒ Ecological, evolutionary & environmental sciences

For a reference copy of the document with all sections, see nature.com/documents/nr-reporting-summary-flat.pdf

# Ecological, evolutionary & environmental sciences study design

All studies must disclose on these points even when the disclosure is negative.

| | |
|---|---|
| Study description | To quantify how different thermal regimes affect investment in gonads and development of gametes in male and female urchins, we first conducted a 10-week experiment in which 300 animals were incubated in replicate 350L mesocosms that simulated El Niño (N = 4 mesocosms, 60 animals per treatment) or La Niña (N = 4 mesocosms, 60 animals) conditions based on historical, empirical benthic temperature time series from Scripps Pier in La Jolla, California (trends: Figure 1B, map: Figure 1C) that coincide with historical collapses in larval supply in Southern California. We paired these treatments with a range of fixed temperature incubations (10, 13, 16, 17, 18, 20 degrees C, N = 2 mesocosms, 30 animals per treatment), two of which matched the mean temperature of the El Niño (20 degrees C) and La Niña (16 degrees C) (Figure 1). We chose this benthic time series rather than satellite-derived sea surface temperature information because sea surface temperatures can be markedly different than temperatures experienced at depth by benthic organisms55. Experiments were conducted at the Marna Lab at the Hakai Institute's Quadra Island Ecological Observatory in Heriot Bay, British Columbia due to availability of sophisticated seawater systems for precisely controlled and replicated temperature manipulations. However, animals in this first experiment were sourced from populations found in Ucluelet, British Columbia that rarely if ever experience the high temperatures commonly observed in southern California. Thus, we also conducted a second experiment in which animals were sourced from warm and cool populations and compared responses of urchins to different thermal treatments.<br><br>To quantify the degree to which animal source location affected results, we conducted a second 12-week experiment comparing animals from the warmer Southern California bight including San Diego and Santa Barbara with the cool upwelling region of Sonoma County. In this experiment, we exposed animals to the same heatwave treatments (21-18 degrees C), 10 degrees C, and 20 degrees C in a split plot design.<br><br>For both experiments we used the same general methodologies (food source, feeding regime, containers) but in different settings (Quadra Island Ecological Observatory in experiment 1 and Bodega Marine Lab in experiment 2). |
| Research sample | Individual male or female purple sea urchins (Strongylocentrotus purpuratus) from Ucluelet, British Columbia, Canada (48.94̊N, 125.56̊W), Bodega Marine Laboratory with wild animals collected from Stillwater Cove (38.546455, -123.298284), Mohawk Reef |

(34.394139, -119.729361) and Point Loma (32.711484, -117.272601). Ages unknown.

| | |
|---|---|
| Sampling strategy | Sample sizes were determined based on the maximum number of animals estimated to be safely contained within each mesocosm system. |
| Data collection | At the end of the first experiment, we measured all individuals to test for changes in height and diameter (using precision digital calipers) and wet mass (to the nearest 0.1 g). Full growth measurements are reported by Spindel et al. (2023). Animals were then sacrificed to measure gonad and histological properties. After opening urchin tests, we immediately removed gonads for sampling. We excised one gonad from each animal for histological analyses; a second gonad was excised and carefully weighed to the nearest 0.01 g. Using a clean, sterile scalpel we excised an approximately 2 mm cross section from the first gonad which we immediately placed in a histological cassette, preserved in Hartmann's fixative for 24 hours, and transferred to 70% EtOH. Preserved gonads were embedded in paraffin, sliced, stained using eosin and hematoxylin, and mounted. We assessed gonad samples for sex and developmental stage using four visual subsections and the entire sample collectively to ensure agreement among subsamples. Histological slides were scored on a scale of I to IV, where representative stages are depicted in Figure 2. For the second experiment, we only report analyses of histological data in this paper. |
| Timing and spatial scale | Experiment 1 ran from September to December 2021 at the Quadra Island Ecological Observatory in British Columbia, Canada and Experiment 2 ran from September to December 2023 at the Bodega Marine Laboratory in California, USA. |
| Data exclusions | No data that were planned for the present study were excluded. |
| Reproducibility | No attempts to repeat the experiment except those described within the present study were attempted. However, qualitatively similar results were obtained for two experiments conducted here. |
| Randomization | Animals were randomly assigned to treatments after pit-tagging in each study |
| Blinding | Histological samples were scored blind where treatments associated with slides and images were not divulged to those scoring. |

Did the study involve field work? ☐ Yes ☐ No

## Field work, collection and transport

| | |
|---|---|
| Field conditions | N/A |
| Location | Ucluelet, British Columbia, Canada (48.94̊N, 125.56̊W), Bodega Marine Laboratory with wild animals collected from Stillwater Cove (38.546455, -123.298284), Mohawk Reef (34.394139, -119.729361) and Point Loma (32.711484, -117.272601) |
| Access & import/export | For the first experiment, we collected sea urchins by hand on SCUBA in the vicinity of Ucluelet, British Columbia, Canada (48.94̊N, 125.56̊W) from a depth of 7-8 m relative to mean low tide in September 2021 and transported them to the Marna Lab via truck in seawater filled coolers with bubblers in less than 24 hours. Urchins were collected on DFO permit XR 275 2021. We transferred sea urchins to flow-through sea tables and allowed them to recover for a period of one week before placing animals into the mesocosm system. We selected healthy individuals within a constrained size range for incubations (n = 300, mean test diameter = 56.09 mm, range test diameter = 42.12 – 69.46 mm). Finally, we assigned animals to mesocosms at random at ambient temperature and exposed each assigned mesocosm to a temperature ramp, where the ramp reached target temperatures after two weeks from the initial incoming, ambient temperature (mean across all tanks of 13.3℃, SD = 0.3℃) to avoid thermal shock. Once initial target temperatures were reached, they were maintained or, for the variable treatments, were manually adjusted daily in the AM (~8am each day) as needed by 0.5 oC increments in a scheduled manner to match historical mean El Niño and La Niña daily temperature trends shown in Figure 1B.<br>For the second experiment, we collected animals from three locations in California including Stillwater Cove in Sonoma County, Mohawk Reef in Santa Barbara, and Point Loma in San Diego using SCUBA from 3-5 meters mean low water. Urchins were dry transported layered between kelp and transferred via ground transportation and into the ambient flow through sea water tanks at Bodega Marine Lab within 24 hours of collection. Temperature ramps and acclimation periods were manually adjusted in 1ºC increments. |
| Disturbance | N/A |

# Reporting for specific materials, systems and methods

We require information from authors about some types of materials, experimental systems and methods used in many studies. Here, indicate whether each material, system or method listed is relevant to your study. If you are not sure if a list item applies to your research, read the appropriate section before selecting a response.

## Materials & experimental systems

| n/a | Involved in the study |
|---|---|
| ☒ | ☐ Antibodies |
| ☒ | ☐ Eukaryotic cell lines |
| ☒ | ☐ Palaeontology and archaeology |
| ☐ | ☒ Animals and other organisms |
| ☒ | ☐ Clinical data |
| ☒ | ☐ Dual use research of concern |
| ☒ | ☐ Plants |

## Methods

| n/a | Involved in the study |
|---|---|
| ☒ | ☐ ChIP-seq |
| ☒ | ☐ Flow cytometry |
| ☒ | ☐ MRI-based neuroimaging |

# Animals and other research organisms

Policy information about studies involving animals; ARRIVE guidelines recommended for reporting animal research, and Sex and Gender in Research

**Laboratory animals**

Strongylocentrotus purpuratus

**Wild animals**

For the first experiment, we collected 300 sea urchins by hand on SCUBA in the vicinity of Ucluelet, British Columbia, Canada (48.94˚N, 125.56˚W) from a depth of 7-8 m relative to mean low tide in September 2021 and transported them to the Marna Lab via truck in seawater filled coolers with bubblers in less than 24 hours. We transferred sea urchins to flow-through sea tables and allowed them to recover for a period of one week before placing animals into the mesocosm system.

For the second experiment, we collected animals from three locations in California including Stillwater Cove in Sonoma County, Mohawk Reef in Santa Barbara, and Point Loma in San Diego using SCUBA from 3-5 meters mean low water. Urchins were dry transported layered between kelp and transferred via ground transportation and into the ambient flow through sea water tanks at Bodega Marine Lab within 24 hours of collection.

**Reporting on sex**

Sex was directly considered in the analyses and reported in the text and in the data.

**Field-collected samples**

For the first experiment, we placed urchins in a custom-built array of twenty replicated 214 L [90(L) x 59.5(W) x 40(H) cm] acrylic mesocosms supplied with flow-through UV sterilized and filtered seawater (Figure 3). Each mesocosm was capable of independent control of temperature and animals were provided a lighting regime for all mesocosms using LED fixtures (Aquamaxx, CA, USA) programmed to provide 10L:14D with two-hour linear light intensity transition periods for dawn and dusk (0-100% from 07:00 to 09:00 “dawn”, and 100-0% from 17:00 to 19:00 “dusk”). Each mesocosm independently maintained temperature treatments using a heat exchanger fitted with a titanium coil regulated by a dual stage digital temperature controller (Resolution = 0.1?, Dwyer Instruments, LLC.©, Michigan City, IN, USA). The mesocosm system employed central cooling (Aermec Mits Airconditioning Inc., Mississauga, ON, Canada) and heating (boiler array, Viessmann Manufacturing Company Inc., Warwick, RI, USA) to supply independent heat exchangers with on-demand cold and warm glycol loops for down- and up-regulation of water temperature, respectively. We manually checked and re-calibrated sensors, as needed, using digital traceable thermometers twice daily to control potential temperature sensor drift. We randomly assigned mesocosms to the specified treatments.
For the second experiment, we placed individuals in a custom-built experimental array at the Bodega Marine Laboratory in which individuals from each population were placed in a split plot design in replicated (N = 4 each 140L - 73 (W) x 66 (H) x 32 (D) cm) acrylic tanks per treatment (Oceans Design Inc). Tanks were fed by sumps fixed with both 0.25hp chillers (Aqualogic Delta Star®) and 1000W heat sticks (TSHTCE-1000S) and temperature controllers to regulate temperature in a partially recirculating flow through system with fresh seawater allocated to each system at a rate of 0.5L/min (approx. 5x turnover per day). Each sump was affixed with protein skimmers, UV filter, and bio-ball filters such that water was first filtered and then UV sterilized upon recirculation. Temperatures were set by hand each day for the heatwave treatments and checked with temperature probes for all treatments. We ran experiments from September 15, 2023, to December 19, 2023.

Animal husbandry
For both experiments, animals were fed on the same feeding regime and the same food as above, also housed in the same density and same trays for consistency. We fed individuals uniform dry pellets combining several macroalgal species formulated for the aquaculture of S. purpuratus (Urchinomics Canada Inc., Halifax, NS, Canada). Animals in mesocosms were fed twice per week and we removed uneaten food and refuse every 72 h. Food rations were determined by trial and error in a prior pilot study to ensure all animals had consistent access to food over time. To optimize access for all mesocosm inhabitants to abundant food, we enclosed subjects and food in aquaculture baskets (two baskets per mesocosm, 7 or 8 animals per basket, Thunderbird Plastics 48 x 33.5 x 10 cm Fish Farm Tray) such that food was always readily accessible, and movement was not impeded. Each animal was supplied approximately 2.7 grams of pelleted food twice per week (either 19 or 21 grams per basket for the baskets with 7 and 8 individuals, respectively in each mesocosm) for the duration of the experiment.

At the end of the first experiment, we measured all individuals to test for changes in height and diameter (using precision digital calipers) and wet mass (to the nearest 0.1 g). Full growth measurements are reported by Spindel et al. (2023)34. Animals were then sacrificed to measure gonad and histological properties.

**Ethics oversight**

No ethical approval was required but permits were used for collection

Note that full information on the approval of the study protocol must also be provided in the manuscript.

## Plants

| | |
|---|---|
| Seed stocks | *Report on the source of all seed stocks or other plant material used. If applicable, state the seed stock centre and catalogue number. If plant specimens were collected from the field, describe the collection location, date and sampling procedures.* |
| Novel plant genotypes | *Describe the methods by which all novel plant genotypes were produced. This includes those generated by transgenic approaches, gene editing, chemical/radiation-based mutagenesis and hybridization. For transgenic lines, describe the transformation method, the number of independent lines analyzed and the generation upon which experiments were performed. For gene-edited lines, describe the editor used, the endogenous sequence targeted for editing, the targeting guide RNA sequence (if applicable) and how the editor was applied.* |
| Authentication | *Describe any authentication procedures for each seed stock used or novel genotype generated. Describe any experiments used to assess the effect of a mutation and, where applicable, how potential secondary effects (e.g. second site T-DNA insertions, mosiacism, off-target gene editing) were examined.* |
